# Clinical outcomes and prognostic factors in bloodstream infections due to extended-spectrum β-lactamase-producing Enterobacteriaceae among patients with malignancy: a meta-analysis

**DOI:** 10.1186/s12941-020-00395-7

**Published:** 2020-11-23

**Authors:** Ai-Min Jiang, Na Liu, Rui Zhao, Hao-Ran Zheng, Xue Chen, Chao-Xin Fan, Rui Zhang, Xiao-Qiang Zheng, Xiao Fu, Yu Yao, Tao Tian

**Affiliations:** 1 Department of Medical Oncology, The First Affiliated Hospital of Xi’an Jiaotong University, Xi’an, Shaanxi 710061 People’s Republic of China; 2 Department of Nutrition and Food Hygiene, School of Public Health, Tongji Medical College, Huazhong University of Science and Technology, Wuhan, China

**Keywords:** Bloodstream infection, Extended-spectrum β-lactamase, ESBL-PE, Malignancy, Mortality, Meta-analysis

## Abstract

**Background:**

The colonization of Extended-spectrum β-lactamase-producing Enterobacteriaceae (ESBL-PE) in bloodstream infections (BSIs) has been increased dramatically worldwide, and it was associated with worse clinical outcomes in patients with malignancy. We performed the meta-analysis to investigate the prognosis and risk factors in BSIs caused by ESBL-PE in oncological patients.

**Methods:**

PubMed, EMBASE, and Cochrane Library were searched for related studies. All-cause mortality was considered as the primary outcome. Subgroup analyses, meta-regression analyses, and sensitivity analysis were used to investigate heterogeneity and reliability in results.

**Results:**

6,729 patients from 25 studies were eligible. Six studies enrolled oncological patients with BSIs caused by ESBL-PE only, while 19 studies both enrolled ESBL-PE and non-ESBL-PE infections. The results showed that BSIs caused by ESBL-PE in patients with malignancy was associated with higher mortality than non-ESBL-PE infections (RR = 2.21, 95% CI: 1.60–3.06, *P* < 0.001), with a significant between-study heterogeneity (*I*^*2*^ =78.3%, *P* < 0.001). Subgroup analyses showed that children (RR = 2.80, 95% CI: 2.29–3.43, *P* < 0.001) and hematological malignancy (RR = 3.20, 95% CI: 2.54–4.03, *P* < 0.001) were associated with a higher mortality. Severe sepsis/ septic shock, pneumonia, and ICU admission were the most common predictors of mortality.

**Conclusions:**

Our study identified that BSIs caused by ESBL-PE in patients with malignancy were associated with worse clinical outcomes compared with non-ESBL-PE infections. Furthermore, children and hematological malignancy were associated with higher mortality. Severe sepsis/ septic shock, pneumonia, and ICU admission were the most common predictors of mortality.

## Background

In recent years, the incidence of bloodstream infections (BSIs) caused by Extended-spectrum β-lactamase-producing Enterobacteriaceae (ESBL-PE) has been increasing over time all over the world [1]. There is a growing body of evidence to show that BSIs caused by ESBL-PE are more worrisome in clinical practice. Extended-spectrum β-lactamases (ESBLs) mediates resistance to a wide variety of antibiotics, including third-generation cephalosporins, aminoglycosides, and quinolones. Furthermore, most of empirical antimicrobial regimens can not cover these pathogens [2, 3]. Therefore, the antimicrobial therapeutic regimens are often limited in these infections [1].

Patients with malignancy are more vulnerable to developing severe infection, including those caused by ESBL-PE since they are more likely to be immunocompromised due to chemotherapy, radiotherapy, surgery, invasive procedures, malnutrition, and malignancy itself [4, 5]. As a result, these infections have become significant therapeutic challenges for clinicians due to delayed initiation of chemotherapy, reduced standard dosage, prolonged hospitalization, increased financial burden on healthcare, and raised severe morbidity and mortality [3, 6]. Therefore, rapid initiation of appropriate antibiotic therapy is pivotal for oncological patients with BSIs caused by ESBL-PE, [4] while inappropriate empirical antibiotic treatment is associated with worse outcomes and survival [3].

Previous meta-analyses have investigated the prevalence of BSIs caused by ESBL-PE in patients with malignancy [7, 8]. However, there was no further analysis of clinical outcomes and risk factors in these populations. Therefore, we conducted this study to assess the prognosis and risk factors of BSIs due to ESBL-PE in patients with malignancy and provide updated evidence via meta-analysis.

## Methods

### Search strategy

Our meta-analysis was based on the Preferred Reporting Items for Systematic Reviews and Meta-Analyses (PRISMA) guidelines [9]. We conducted an overall literature retrieval for PubMed, EMBASE, and Cochrane Library published up to 10 December 2019. Both MeSH terms and free words were used to search for title/ abstract. Our search terms were: “(ESBL OR (extended-spectrum beta-lactamase) OR (extended-spectrum β-lactamase)) AND (tumor OR neoplasia OR malignancy OR cancer OR carcinoma OR sarcoma OR leukemia OR leukaemia OR lymphoma OR hematolog* OR haematolog* OR oncolog*)”. We manually screened other relevant studies and reference lists. The search was performed independently by two investigators (AM Jiang and N Liu).

### Study selection

Studies were considered as eligible based on the following criteria: [1] population: patients with solid or hematological malignancies; [2] intervention (exposure): BSIs caused by ESBL-PE; [3] comparison: BSIs caused by non-ESBL-PE; [4] outcome: the mortality of BSIs. Literature that satisfied the following criteria were excluded: [1] letters, case reports, editorials, expert opinion or reviews without original data; [2] overlapping or duplicate data; [3] incomplete data about outcomes; [4] not English literatures; [5] the sample size of BSIs caused by ESBL-PE in oncological patients less than 10; [6] studies only focusing on risk factors for ESBL-PE infections.

### Data extraction and quality assessment

Two investigators (AM Jiang and N Liu) independently extracted the data using a standardized approach. Any disagreement in the study selection and data extraction phases was resolved through discussion with the third investigator (R Zhao). The following data information was retrieved from each article: first author’s name, year of publication, country, study population, infection type of BSIs, the total number of screened subjects, the total number of ESBL-PE caused BSIs, the total number of BSIs caused death and ESBL-PE BSIs caused death, and ESBL detection method. The data was extracted from texts or tables in articles. Newcastle Ottawa Quality Assessment Scale (NOS) was used in our research to assess the quality of selected studies [10]. The scale included three aspects: selection, comparability, and outcome. Studies that scored more than five were considered of high quality.

### Definitions and study outcomes

Neutropenia was defined as an absolute neutrophil count of < 500 neutrophils/mm^3^ [11].

Empirical antibiotics treatment was considered inappropriate once the antibiotics could not suppress the activity of the isolated pathogens according to the results of antimicrobial susceptibility tests during the first 24 h after the blood culture was obtained [11].

The all-cause mortality at the end of the study and the predictors in BSIs due to ESBL-PE in patients with malignancy were the primary outcomes in the study [12].

### Statistical analysis

The RRs and 95% CIs for mortality were calculated to assess the outcomes of BSIs caused by ESBL-PE in oncological patients. All results were depicted as forest plots. Heterogeneity was assessed using Cochran’s Q test and the *I*^*2*^ statistic test. When the heterogeneity was statistically significant (*P* < 0.05 and *I*^*2*^ > 50%), a random-effects model was applied to obtain the pooled RRs; otherwise, a fixed-effects model was performed. Subgroup analyses and meta-regression analyses were conducted to explore the sources of heterogeneity. We also performed a sensitivity analysis to evaluate the quality and stability of results by omitting one study in each turn. Begg’s test and Egger’s test were used to assess the publication bias. Statistical tests were two-tailed at the significance level of *P* < 0.05. All analyses were used with STATA V.14.0 (Stata Corporation, College Station, TX).

## Results

### Study characteristics and quality assessment

**Fig. 1:**
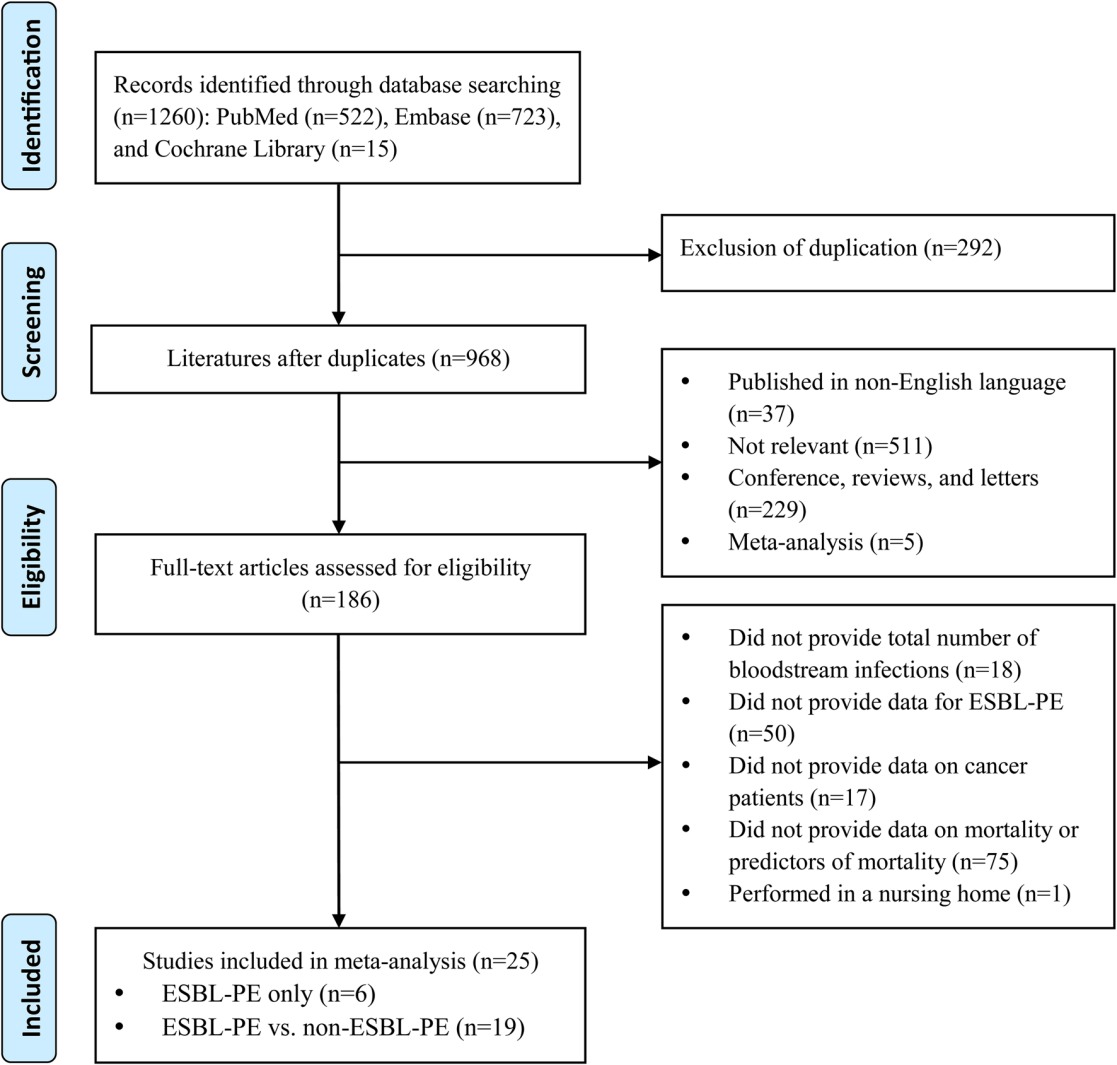
Flow chart of the eligible studies for meta-analysis

Our literature search identified 1,260 studies. After excluding repeated records and the initial screening based on titles and abstracts, 25 articles were eligible in this study. Of these, six studies enrolled oncological patients with BSIs caused by ESBL-PE only, while 19 studies both enrolled ESBL-PE and non-ESBL-PE infections. Of the 25 studies, there were eight prospective cohort studies, 14 retrospective cohort studies, and three case–control studies. All included studies were published between 2009 and 2019, and there were six studies published in 2019, accounting for 24%. There were 15 studies conducted in Asia, seven studies conducted in Europe, and three studies were conducted in North America. The detailed flow chart of the study selection process was described in Fig. 1. Table 1 summarized the characteristics of 25 selected studies. The majority of the included studies had a NOS score of more than 5 points, and 21 studies included in this article were high-quality studies. Additional file 1: Table S1 presented the results of the quality assessment.

**Table 1:** Characteristics of the studies and their populations included in the review

First author	Design	Year	Country	Population	Infection type—bacteria	No. of screened	No. of ESBL-PE BSIs (%)	No. of BSIs caused death	No. of ESBL-PE BSIs caused death (%)	ESBL detection method
ESBL-PE only										
Wang [31]	MC retrospective cohort	2011	Taiwan, China	Adults with solid or hematological malignancy	*E. coli* and Klebsiella	351	113 (32.2%)	35	35 (31.0%)	MicroScan
Wu [15]	SC prospective cohort	2012	Taiwan, China	Adults with solid or hematological malignancy	*E. coli*	97	39 (40.2%)	10	10 (25.6%)	Disk diffusion
Kang [17]	MC retrospective cohort	2013	Korea	Adults with solid malignancy	*E. coli*	92	36 (39.1%)	6	6 (16.7%)	Microdilution or disk diffusion
Gudiol [29]	MC retrospective cohort	2017	Spain	Adults with acute leukemia and neutropenia	*E. coli*, Klebsiella, and *Enterobacter cloacae*	NR	425	65	65 (15.3%)	Disk diffusion
Cattaneo [23]	MC prospective cohort	2018	Germany	Adults with hematological malignancy	*E. coli*, Klebsiella, and other *Enterobacter spp*	137	61 (44.5%)	10	10 (16.4%)	Microdilution
Benanti [32]	SC retrospective cohort	2019	USA	Adults with hematological malignancy	*E. coli*	NR	103	11	11 (10.7%)	NR
ESBL-PE and non-ESBL-PE										
Trecarichi [13]	SC retrospective cohort	2009	Italy	Adults and children with hematological malignancy	*E. coli*	107	26 (24.3%)	13	11 (42.3%)	Disk diffusion
Gudiol [27]	SC prospective cohort	2010	Spain	Adults and children with solid or hematological malignancy	*E. coli*	531	17 (3.2%)	29	6 (35.3%)	MicroScan
Cornejo-Juarez [40]	Case–control study	2012	Mexico	Adults and children with hematological malignancy	*E. coli*	670	100 (14.9%)	102	51 (51%)	MicroScan
Kang [14]	MC retrospective cohort	2012	Korea	Adults with hematological malignancy	*E. coli* and Klebsiella	142	29 (20.4%)	29	13 (44.8%)	Microdilution or disk diffusion
Ha [16]	SC retrospective cohort	2013	Korea	Adults with solid or hematological malignancy	*E. coli*	350	95 (27.1%)	52	21 (22.1%)	Disk diffusion
Kim [18]	SC retrospective cohort	2013	Korea	Adults with hematological malignancy and FN	*E. coli* and Klebsiella	96	23 (24.0%)	8	4 (17.4%)	MicroScan
Metan [34]	SC retrospective cohort	2013	Turkey	Adults and children with hematological malignancy	*E. coli*, Klebsiella, and *Enterobacter cloacae*	154	40 (26.0%)	30	5 (12.5%)	NR
Bodro [19]	SC prospective cohort	2014	Spain	Adults with solid or hematological malignancy	Klebsiella and other *Enterobacter spp*	392	19 (4.8%)	18	4 (21.1%)	Disk diffusion
Kim [28]	SC prospective cohort	2014	Korea	Adults and children with solid or hematological malignancy	*Enterobacter spp*	203	31 (15.3%)	34	6 (19.4%)	Disk diffusion
Han [20]	SC retrospective cohort	2015	Korea	Children with solid or hematological malignancy and FN	*E. coli* and Klebsiella	59	21 (35.6%)	3	1 (4.8%)	VITEK®2 automated system
Cattaneo [21]	MC prospective cohort	2016	Italy	Adults with acute leukaemia	*Enterobacter spp*	433	36 (8.3%)	37	5 (13.9%)	Microdilution or disk diffusion
Ma [22]	SC retrospective cohort	2017	China	Adults with hematological malignancy	*E. coli*	168	97 (57.7%)	21	15 (15.5%)	Disk diffusion
Çeken [24]	SC retrospective cohort	2018	Turkey	Adults with solid or hematological malignancy	*E. coli* and Klebsiella	122	70 (57.4%)	31	23 (32.9%)	VITEK®2 automated system
Islas-Muñoz [25]	SC prospective cohort	2018	Mexico	Adults with solid or hematological malignancy	*E. coli*, Klebsiella, and *Enterobacter spp*	496	123 (24.8%)	89	37 (30.1%)	Disk diffusion
Ben-Chetrit [42]	SC retrospective cohort	2019	Israel	Adults with solid or hematological malignancy	*E. coli* and Klebsiella	88	26 (19.5%)	17	6 (7.5%)	NR
Isendahl [26]	Case–control study	2019	Sweden	Adults and children with solid or hematological malignancy	*E. coli* and Klebsiella	945	238 (25.2%)	107	45 (18.9%)	NR
Kim [35]	SC prospective cohort	2019	Korea	Adults with solid or hematological malignancy and FN	*E. coli* and Klebsiella	179	23 (12.8%)	51	8 (34.8%)	MicroScan
Namikawa [30]	Case–control study	2019	Japan	Adults with solid or hematological malignancy	*E. coli*, Klebsiella, and *Enterobacter spp*	65	42 (64.6%)	13	9 (21.4%)	NR
Zhang [33]	SC retrospective cohort	2019	China	Adults with solid or hematological malignancy	*E. coli*	324	160 (49.3%)	71	39 (24.4%)	VITEK®2 automated system

*ESBL-PE* extended-spectrum β-lactamase-producing Enterobacteriaceae, *BSI* bloodstream infection, *SC* single-center, *MC* multicenter, *NR* not reported, *FN* febrile neutropenia

### Mortality

**Fig. 2:**
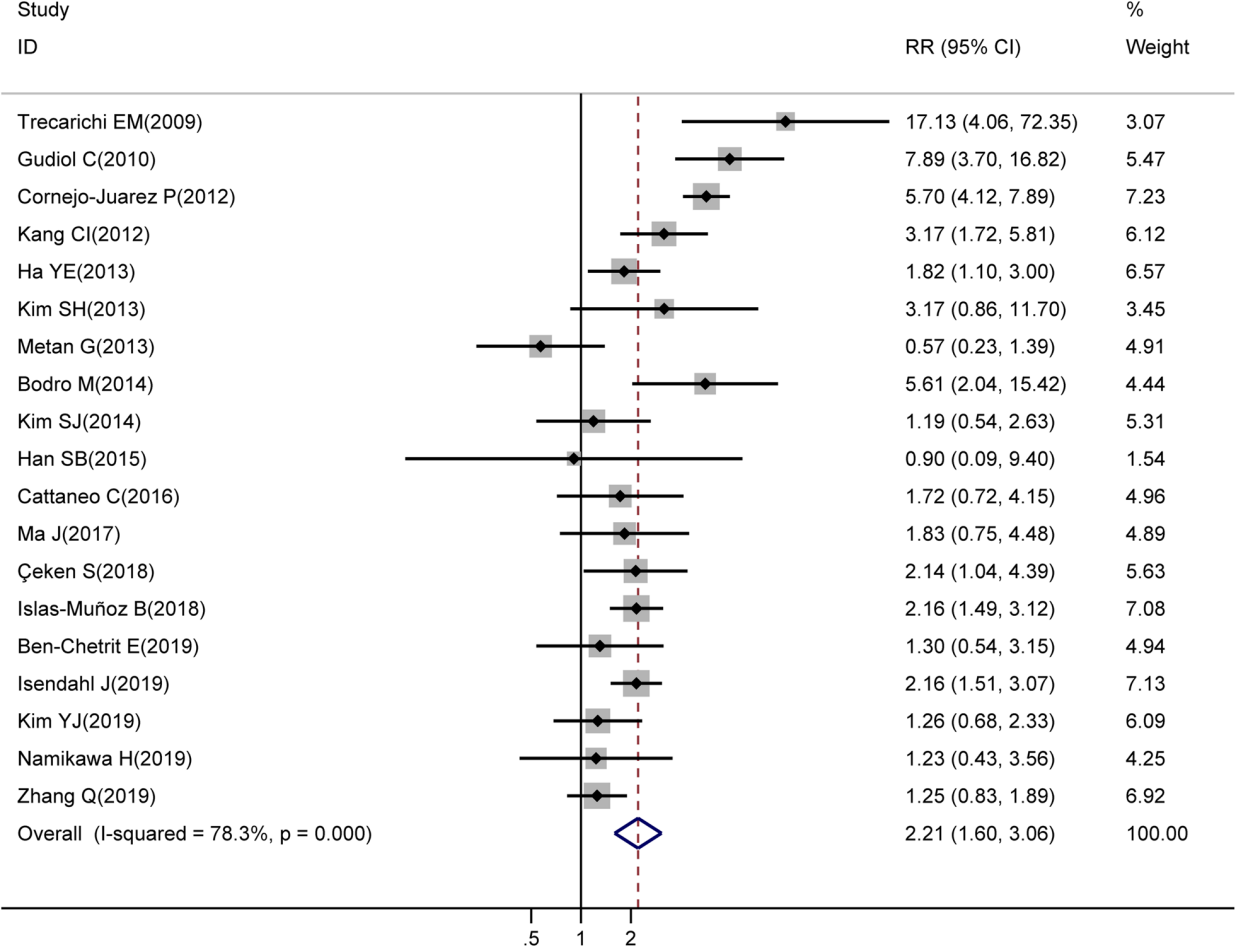
Forest plot of mortality in BSIs due to ESBL-PE among patients with malignancy. *RR* relative risk, *CI* confidence interval, *BSIs* bloodstream infections, Weights are from random-effects analysis. The size of the squares is analogous to the study's weight. Diamonds represent the pooled RRs and their confidence interval

In all the studies, the time of death records was not similar. The majority of studies used 30-day mortality to evaluate the clinical outcomes of BSIs caused by ESBL-PE in patients with malignancy, [13–29] only one study did not report a particular time of death [30]. In studies that only enrolled oncological patients with ESBL-PE infections, the mortality of BSIs varied from 10.7 to 31.0%. However, the mortality of BSIs varied from 4.8 to 51.0% in studies that both enrolled ESBL-PE and non-ESBL-PE infections oncological patients, respectively. We finally included 19 studies that enrolled both ESBL-PE and non-ESBL-PE infected oncological patients into analyses to estimate the mortality of BSIs caused by ESBL-PE in patients with malignancy. The results showed that in patients with malignancy, ESBL-PE infections were associated with a higher mortality risk from BSIs than non-ESBL-PE infections (RR = 2.21, 95% CI: 1.60–3.06, *P* < 0.001) (Fig. 2), with a significant between-study heterogeneity (*I*^*2*^ = 78.3%, *P* < 0.001).

### Subgroup analyses and meta-regression analyses

Subgroup analyses in all selected studies were conducted by study design, region, study population, malignancy type, FN, ESBL detection methods, and NOS score. Most of the subgroups (study design, region, study population, malignancy type, and ESBL detection methods) were consistent with the overall trend and showed statistically significant increases, except for the subgroup without FN, and the subgroup with NOS score < 6. The subgroup analyses suggested that study region was identified as potential sources of the heterogeneity (test for subgroup difference: *P* = 0.014), and the RR of mortality in studies from Asia (RR = 1.49, 95% CI: 1.22–1.82) was lower compared with Europe and North America, with no evidence of heterogeneity (*I*^*2*^ = 27.3%, *P* = 0.177). The detailed information was in Table 2 and Additional file 1: Figure S1.

**Table 2:** Subgroup analysis for meta-analysis of mortality

Variables		No	RR (95% CI)	*I*^*2*^	*P*^*a*^	*P*^*b*^
Design	Retrospective cohort	10	1.72 (1.38–2.14)	62.2%	0.005	0.395
	Prospective cohort	6	2.02 (1.57–2.60)	75.7%	0.001	
	Case–control study	3	3.11(2.47–3.92)	90.2%	< 0.001	
Region	Europe	5	2.79(2.10–3.69)	78.2%	0.001	0.014
	Asia	12	1.49(1.22–1.82)	27.3%	0.177	
	North America	2	3.47 (2.73–4.41)	93.5%	< 0.001	
Population	Adults and children	7	2.80 (2.29–3.43)	88.3%	< 0.001	0.303
	Adults	12	1.80 (1.50–2.15)	28.4%	0.167	
Malignancy type	Hematological	7	3.20 (2.54–4.03)	83.0%	< 0.001	0.355
	Solid or hematological	12	1.81 (1.54–2.14)	60.9%	0.003	
FN	Yes	3	1.48 (0.87–2.53)	0.0%	0.418	0.530
	No	16	2.21 (1.92–2.54)	80.8%	< 0.001	
ESBL detection methods	Disk diffusion	6	2.21 (1.72–2.83)	64.6%	0.015	0.554
	MicroScan	4	3.99 (3.10–5.15)	85.9%	< 0.001	
NOS	< 6	2	0.76 (0.39–1.49)	16.5%	0.274	0.065
	≥ 6	17	2.30 (2.00–2.64)	77.3%	< 0.001	

*RR* relative risk, *CI* confidence interval, *FN* febrile neutropenia, *ESBL* extended-spectrum β-lactamase; *NOS* Newcastle–Ottawa scale

^a^*p* for heterogeneity within each subgroup

^b^*p* for heterogeneity between subgroups with meta-regression analyses

### Sensitivity analysis and publication bias

We then carried out the sensitivity analysis by omitting each study in turn. As summarized in Additional file 1: Figure S2, the pooled RRs and 95% CIs of mortality ranged from 2.03 (1.53–2.68) to 2.36 (1.72–3.25). The results of the sensitivity analysis show that our results are stable and reliable since there were no individual studies influenced the overall results. Begg’s test and Egger’s test showed no evidence of publication bias (*P* = 0.944 for Begg’s test; *P* = 0.538 for Egger’s test, respectively) (Additional file 1: Figure S3).

### Predictors of mortality in BSIs caused by ESBL-PE among patients with malignancy

We then summarized the risk factors for BSIs caused by ESBL-PE in patients with malignancy. It showed that the most commonly studied risk factors for BSIs caused by ESBL-PE in patients with malignancy were age, gender, ESBL production, neutropenia, inadequate initial antimicrobial treatment, ICU admission, intra-abdominal infection, pneumonia, Pitt bacteremia score, severe sepsis/ septic shock, solid tumor, and concurrent corticosteroid therapy. However, metastasis and mechanical ventilation were the least studied variables. We also found that the most common independent risk factors of mortality were severe sepsis/ septic shock, pneumonia, ICU admission, and neutropenia. At the same time, indwelling urinary catheter, [23] pneumonia, [31] Pitt bacteremia score, [32] and severe sepsis/ septic shock [31] were the most common independent risk factors of mortality in studies that only enrolled patients with BSIs caused by ESBL-PE. In studies that both included ESBL-PE and non-ESBL-PE infections, severe sepsis/ septic shock, [14, 16, 18, 19, 22, 24, 28, 33] ICU admission, [14, 19, 27, 33, 34] neutropenia, [13, 14, 24, 35] and pneumonia [14, 16, 21, 28] were the most commonly investigated independent risk factors, respectively. Interestingly, there were only three studies [13, 14, 16] identified that ESBL production was associated with unfavorable outcomes in these patients. The detailed information was in Table 3.

**Table 3:** The most commonly studied characteristics as predictors of mortality in the reviewed studies

Risk factor	ESBL-PE only studies n/N (%)	ESBL-PE vs non- ESBL-PE studies n/N (%)	Total n/N (%)	Identified as independent predictor for mortality
Age	5/6 (83.3)	13/19 (68.4)	18/25 (72.0)	0 + 0
Gender	4/6 (66.7)	11/19 (57.9)	15/25 (60.0)	0 + 1 (22)
CCI	1/6 (16.7)	4/19 (21.1)	5/25 (20.0)	0 + 2 (16,42)
ESBL production	1/6 (16.7)	12/19 (63.2)	13/25 (52.0)	0 + 3 (13,14,16)
Neutropenia	2/6 (33.3)	11/19 (57.9)	13/25 (52.0)	0 + 4 (13,14,24,35)
Inadequate initial antimicrobial treatment	3/6 (50.0)	13/19 (68.4)	16/25 (64.0)	0 + 3 (13,19,25)
ICU admission	2/6 (33.3)	6/19 (31.6)	8/25 (32.0)	0 + 5 (14,19,27,33,34)
Immunosuppressant use	0	3/19 (15.8)	3/25 (12.0)	0 + 1 (14)
Indwelling urinary catheter	2/6 (33.3)	1/19 (5.3)	3/25 (12.0)	1 (23) + 1 (14)
Infecting organism, Klebsiella pneumoniae	0	5/19 (26.3)	5/25 (20.0)	0 + 1 (18)
Intra-abdominal infection	4/6 (66.7)	3/19 (15.8)	7/25 (28.0)	0 + 1 (28)
Mechanical ventilation	0	2/19 (10.5)	2/25 (8.0)	0 + 1 (16)
Metastasis	0	1/19 (5.3)	1/25 (4.0)	0 + 1 (33)
Organ failure	1/6 (16.7)	2/19 (10.5)	3/25 (12.0)	0 + 1 (33)
Pneumonia	1/6 (16.7)	5/19 (26.3)	6/25 (24.0)	1 (31) + 4 (14,16,21,28)
Pitt bacteremia score	4/6 (66.7)	4/19 (21.1)	8/25 (32.0)	1 (32) + 2 (14,42)
Severe sepsis/ septic shock	3/6 (50.0)	11/19 (57.9)	14/25 (56.0)	1 (31) + 8 (14,16,18,19,22,24,28,33)
Solid tumor	3/6 (50.0)	8/19 (42.1)	11/25 (44.0)	0 + 1 (27)
Simultaneous corticosteroid therapy	0	8/19 (42.1)	8/25 (32.0)	0 + 2 (19,27)

*ESBL-PE* extended-spectrum β-lactamase-producing Enterobacteriaceae, *CCI* Charlson Index Score, *ICU* intensive care unit

Refers to the variables for which data were reported in the individual studies

## Discussion

Over the past ten years, the colonization and prevalence of ESBL-PE infections have continued to increase rapidly all over the world,[36] and these infections generally associated with worse clinical outcomes, prolonged hospitalization, extra healthcare burden, and delayed initiation of treatment for malignancy [3]. Patients with malignancy are more easily to develop BSIs caused by ESBL-PE since oncological patients are easily immunocompromised due to a series of mechanisms as mentioned before [5]. Therefore, timely and appropriate empirical antimicrobial therapeutic regimen is pivotal for patients with malignancy who developed BSIs caused by ESBL-PE [5].

In this meta-analysis, we included 19 studies that both enrolled oncological patients with ESBL-PE and non-ESBL-PE infections, and the results showed that the mortality in BSIs caused by ESBL-PE among patients with malignancy was higher compared with non-ESBL-PE infections (RR = 2.21, 95% CI: 1.60–3.06, *P* < 0.001). Consistent with our findings, Trecarichi EM et al. reported that ESBL-PE caused BSIs in patients with malignancy was associated with high mortality compared with non-ESBL-PE infections [13, 14, 16]. This result suggests that we should think highly of BSIs caused by ESBL-PE in patients with malignancy during hospitalization, and the rapid initiation of antibiotics treatment should be considered as early as possible once it was recognized.

The results of the subgroup analyses showed that the mortality of ESBL-PE BSIs varies from different regions. We found that the mortality in North America and Europe was higher than in Asia. It could be explained by the fact that the majority of studies were conducted in Asia, and the study region was confirmed as a source of heterogeneity after further meta-regression analyses. Alevizakos M et al. reported that ESBL-PE are the causative agents of approximately 10.0% BSIs among patients with malignancy in Southeast Asia, and it has been associated with increased mortality in these subjects compared with Europe and America [37]. Therefore, more relevant studies need to be included in the future to draw a more reliable conclusion. We also found that children and hematological malignancy were also associated with worse prognosis in BSIs caused by ESBL-PE. It may be attributed to the fact that the included children population were mainly diagnosed with hematological malignancies, which were more vulnerable to develop immunosuppression, prolonged neutropenia, and septic shock [38, 39]. Interestingly, we observed that FN was not associated with higher mortality in oncological patients with BSIs caused by ESBL-PE. This can be explained by the fact that only three studies included patients with FN. Besides, some studies only enrolled a subset of FN patients, but the data were not accessible to analyze [13, 14, 16, 21, 24, 25, 30, 34, 40]. Among these studies, Kang CI et al. reported that FN/ neutropenia was not the risk factor for mortality in BSIs caused by ESBL-PE among patients with malignancy [14, 16, 21, 24]. Hence, the accuracy of the conclusion needs to be further confirmed. The combined RR of sensitivity analysis further confirmed the stability of the results. Besides, the meta-regression analyses also suggested that the study region might be the source of heterogeneity in this meta-analysis, despite other relevant factors such as age, comorbidities, and antimicrobial treatment regimens cannot be analyzed due to lack of relevant data. However, Begg’s test and Egger’s test showed there was no evidence of publication bias in our study.

In our study, approximately 72.0% of studies analyzed the relationship between age and mortality of BSIs caused by ESBL-PE in patients with malignancy. However, there were no studies that identified age as independent predictor. Furthermore, more than 50.0% of studies concluded that severe sepsis/ septic shock, pneumonia, and ICU admission were the most common independent risk factors in mortality. Besides, fewer studies confirmed that neutropenia was more common in patients who died. According to a study conducted by Vardakas KZ et al., they reported that underlying diseases and severity scores were the most commonly identified prognostic factors of mortality in patients with infections due to multi-drug resistant Gram-negative bacteria (MDRGNB) [12]. Similar to the previous study, [12] we also found that severe sepsis/ septic shock was the most common risk factor in mortality. However, only fewer studies concluded that the Pitt bacteremia score and Charlson Index Score (CCI) were more common in patients who died. An interesting finding of our study is that only three studies confirmed ESBL production was an independent risk factor in mortality. Rottier WC et al. reported that ESBL production was associated with higher mortality compared with bacteremia with non-ESBL-PE [41]. This could be due to the small sample size of some studies we included in this study. Therefore, more prospective multicenter studies and clinical trials were urgently needed in the future to provide sufficient evidence.

To our knowledge, this is the first study that evaluated the clinical outcomes and risk factors in BSIs caused by ESBL-PE among patients with malignancy using Meta-analysis. However, our study has several limitations. First, all of the included articles were observational studies and published in English. Besides, high-estimated heterogeneity was observed, which is probably related to different design, study region, and study population. Moreover, some studies included oncological patients with FN, but the data was not available for subgroup analysis. Therefore, the clinical outcomes of BSIs caused by ESBL-PE in these patients should be further validated since these patients are more vulnerable to severe infections. To sum up, clinical conclusions need to be comprehensive assessment in combination with other indicators, and more sample sizes and studies need to be added to verify our results.

## Conclusions

In summary, our study provided a systematic analysis for the prognosis and risk factors of BSIs due to ESBL-PE in oncological patients. Our findings suggested that BSIs caused by ESBL-PE in patients with malignancy were associated with worse clinical outcomes compared with non-ESBL-PE infections. Furthermore, children and hematological malignancy were associated with higher mortality. We also identified that severe sepsis/ septic shock, pneumonia, and ICU admission were the most common predictors of mortality. Large-scale and prospective studies are warranted to verify the results of our study.

## Supplementary information

**Additional file 1: Table S1.** Quality assessment conducted according to the NOS for all included studies. **Figure S1.** Forest plots of mortality in BSIs due to ESBL-PE among patients with malignancy by different subgroups. (**a**) study design; (**b**) region; (**c**) population; (**d**) malignancy type (**e**) FN; (**f**) ESBL detection methods; (**g**) NOS score.** Figure S2.** Sensitivity analysis of mortality in BSIs due to ESBL-PE among patients with malignancy. **Figure S3.** Tests for publication bias. **a** Begg's funnel plot with pseudo 95% confidence limits; **b** Egger's publication bias plot.

## Data Availability

Data sharing is not applicable to this article as no datasets were generated or analyzed during the current study.

## Abbreviations

ESBL-PE: Extended-spectrum β-lactamase-producing Enterobacteriaceae
BSI: Bloodstream infection
SC: Single-center
MC: Multicenter
NR: Not reported
FN: Febrile neutropenia
RR: Relative risk
CI: Confidence interval
NOS: Newcastle–Ottawa scale
CCI: Charlson Index Score
ICU: Intensive care unit
