# Supplementary material for: Clinical outcomes and prognostic factors in bloodstream infections due to extended-spectrum β-lactamase-producing Enterobacteriaceae among patients with malignancy: a meta-analysis
Source: Ann Clin Microbiol Antimicrob. 2020 Nov 23;19:53. doi: 10.1186/s12941-020-00395-7 (PMC7685587; doi:10.1186/s12941-020-00395-7)
Supplement: Supplementary file 1 — Additional file 1: Table S1. Quality assessment conducted according to the NOS for all included studies. Figure S1. Forest plots of mortality in BSIs due to ESBL-PE among patients with malignancy by different subgroups. (a) study design; (b) region; (c) population; (d) malignancy type (e) FN; (f) ESBL detection methods; (g) NOS score. Figure S2. Sensitivity analysis of mortality in BSIs due to ESBL-PE among patients with malignancy. Figure S3. Tests for publication bias. a Begg's funnel plot with pseudo 95% confidence limits; b Egger's publication bias plot. [file 12941_2020_395_MOESM1_ESM.docx]

**Table S1**

Quality assessment conducted according to the NOS for all included studies.

| Study | Selection | Comparability | Outcome | Quality Score |
| --- | --- | --- | --- | --- |
| Trecarichi EM | ★★★ | ★★ | ★★★ | **8** |
| Gudiol C | ★★★ | ★★ | ★★★ | **8** |
| Wang SS | ★★★ | _ | ★★★ | **6** |
| Cornejo-Juarez P | ★★★ | ★ | ★★★ | **7** |
| Kang CI | ★★★★ | ★ | ★★★ | **8** |
| Wu UI | ★★ | _ | ★★★ | **5** |
| Ha YE | ★★★ | ★★ | ★★★ | **8** |
| Kang CI | ★★★ | _ | ★★★ | **6** |
| Kim SH | ★★★ | ★ | ★★★ | **7** |
| Metan G | ★★ | _ | ★★ | **4** |
| Bodro M | ★★★ | ★★ | ★★★ | **8** |
| Kim SJ | ★★★ | ★ | ★★★ | **7** |
| Han SB | ★★★ | ★ | ★★★ | **7** |
| Cattaneo C | ★★★ | _ | ★★★ | **6** |
| Gudiol C | ★★★ | _ | ★★★ | **6** |
| Ma J | ★★★ | ★★ | ★★★ | **8** |
| Cattaneo C | ★★★ |  | ★★★ | **6** |
| Çeken S | ★★★ | ★★ | ★★★ | **8** |
| Islas-Muñoz B | ★★★ | _ | ★★★ | **6** |
| Benanti GE | ★★ | _ | ★★ | **4** |
| Ben-Chetrit E | ★★★ | ★ | ★★★ | **7** |
| Isendahl J | ★★★★ | _ | ★★★ | **7** |
| Kim YJ | ★★★ | ★ | ★★ | **6** |
| Namikawa H | ★★★ | _ | ★★ | **5** |
| Zhang Q | ★★★ | ★★ | ★★★ | **8** |

★represents points of score.


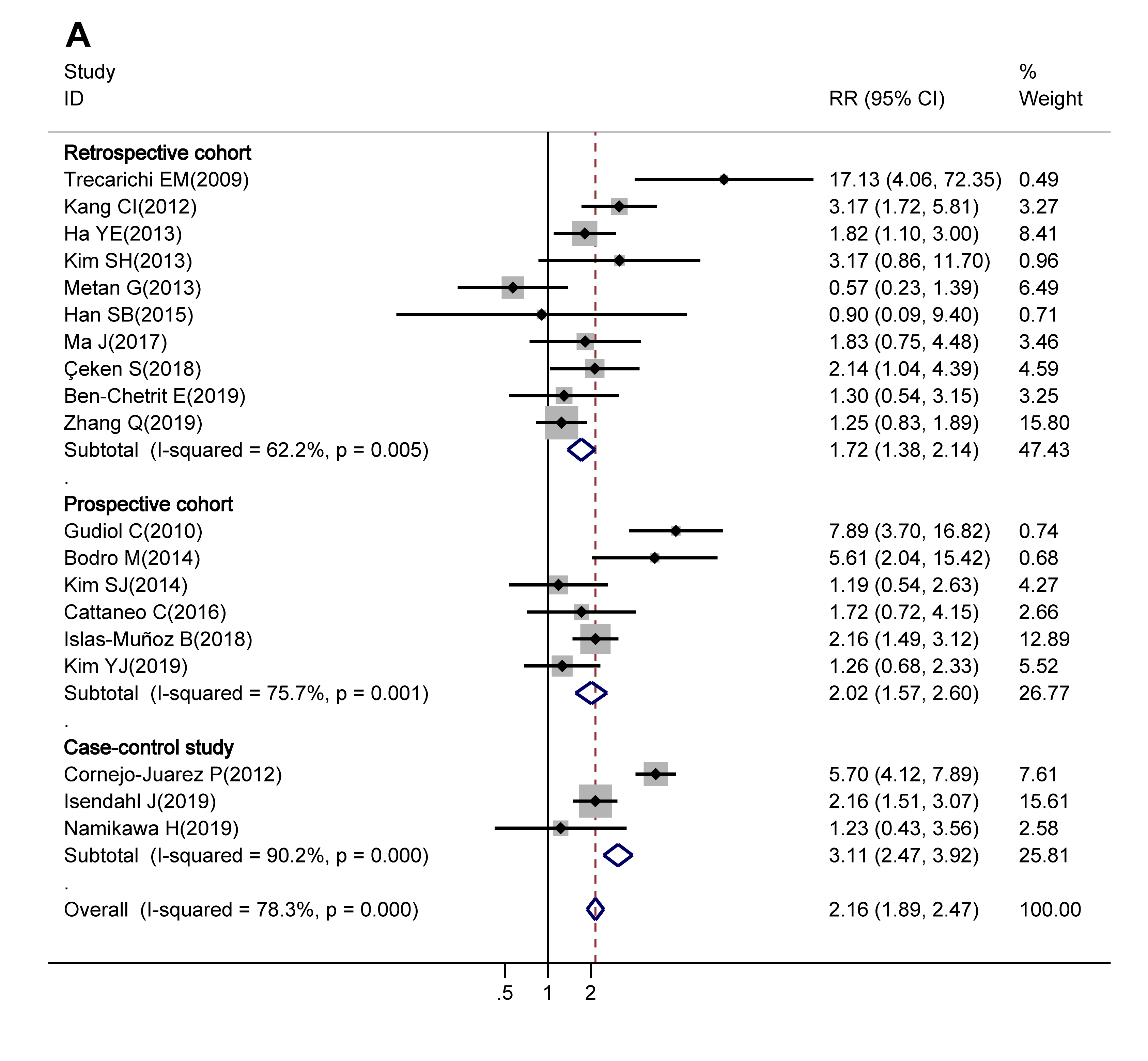


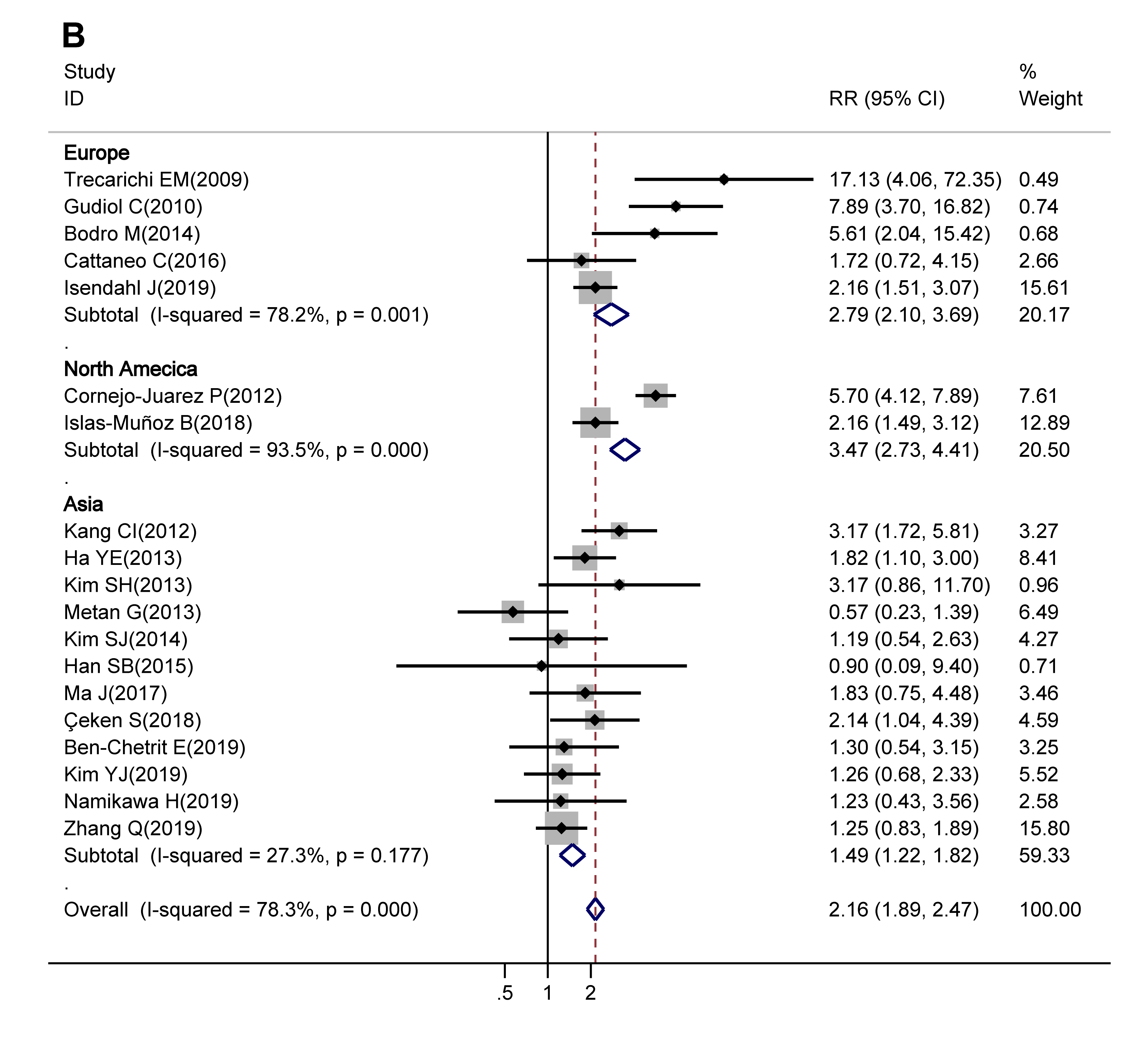


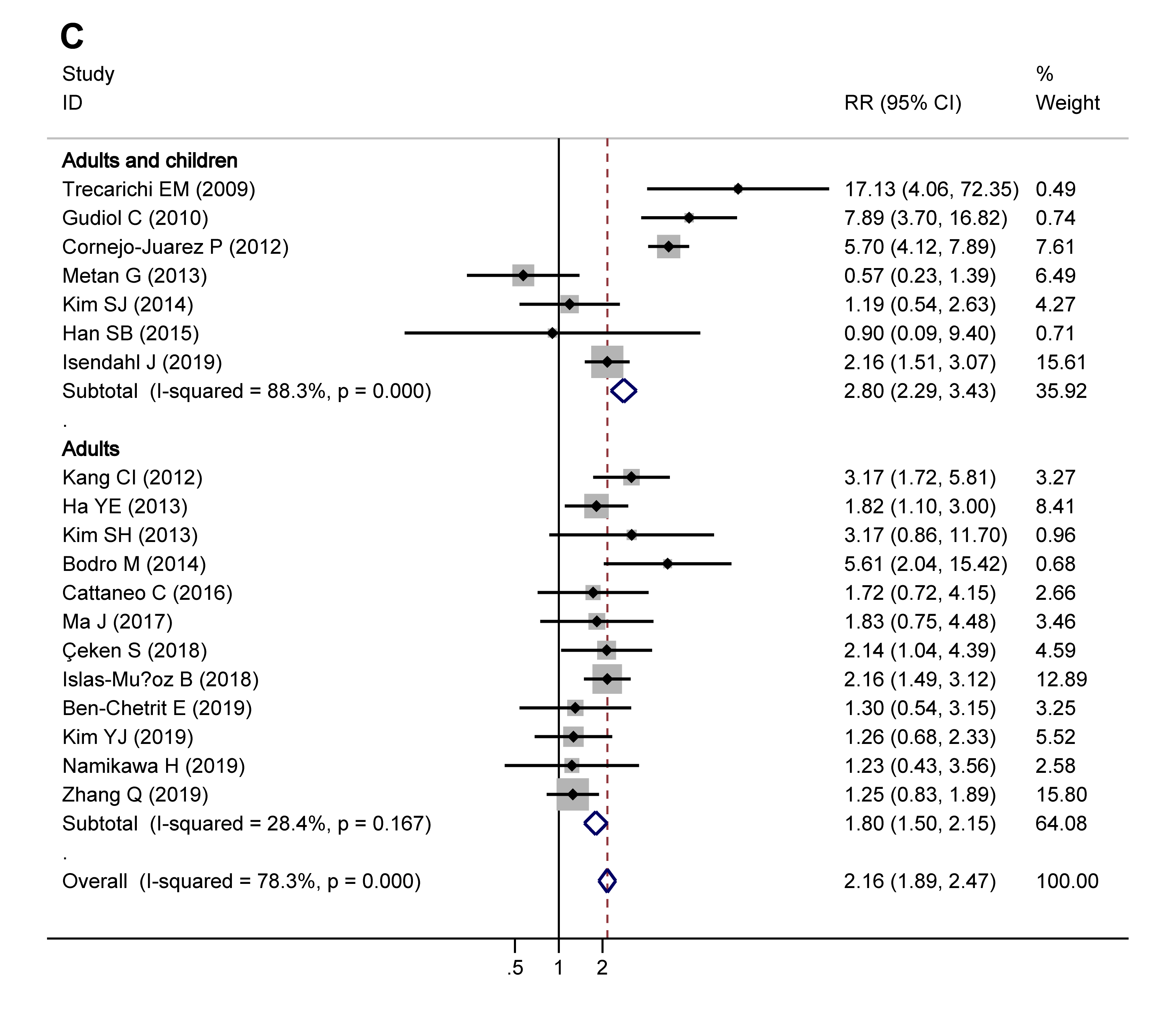


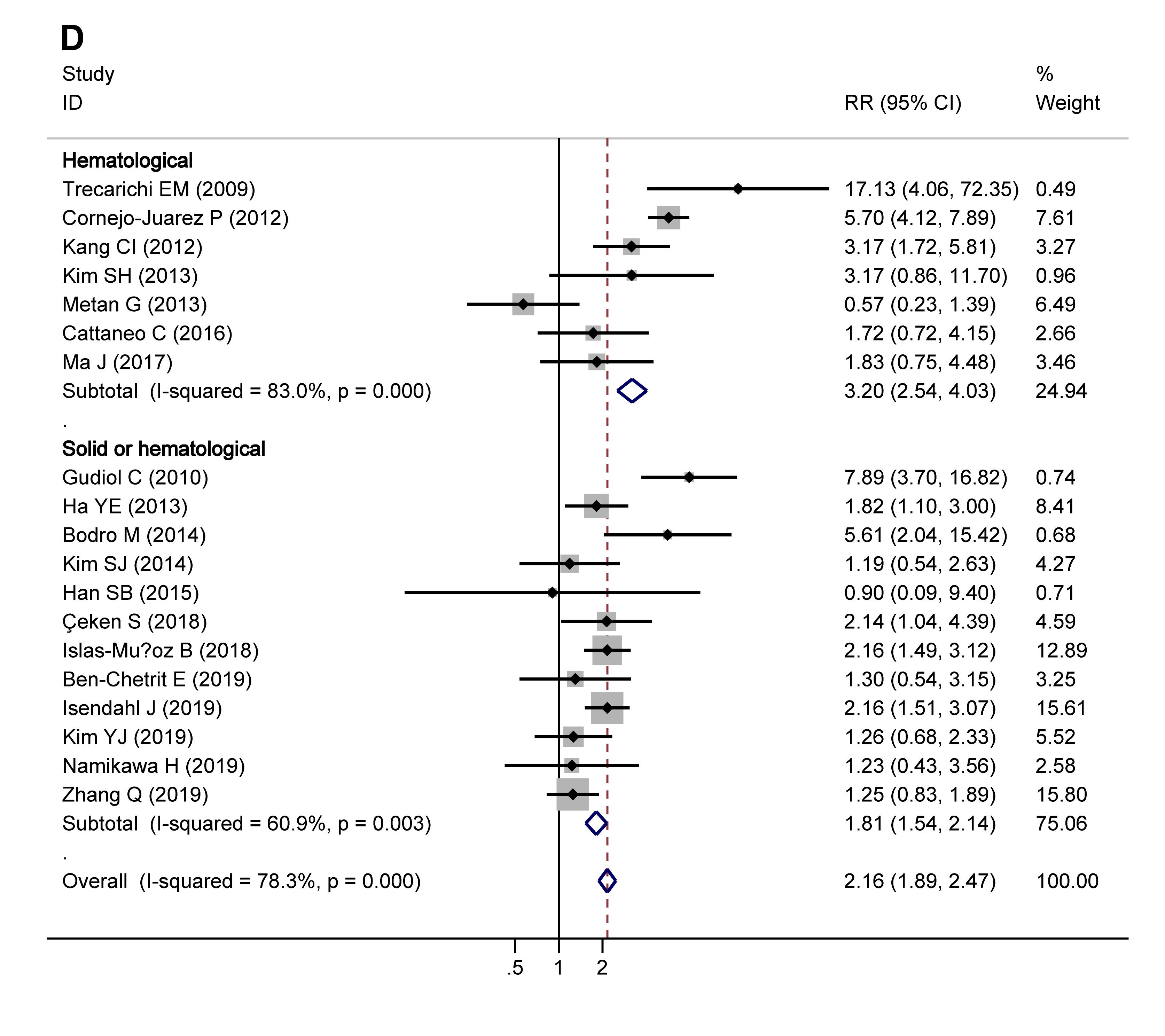


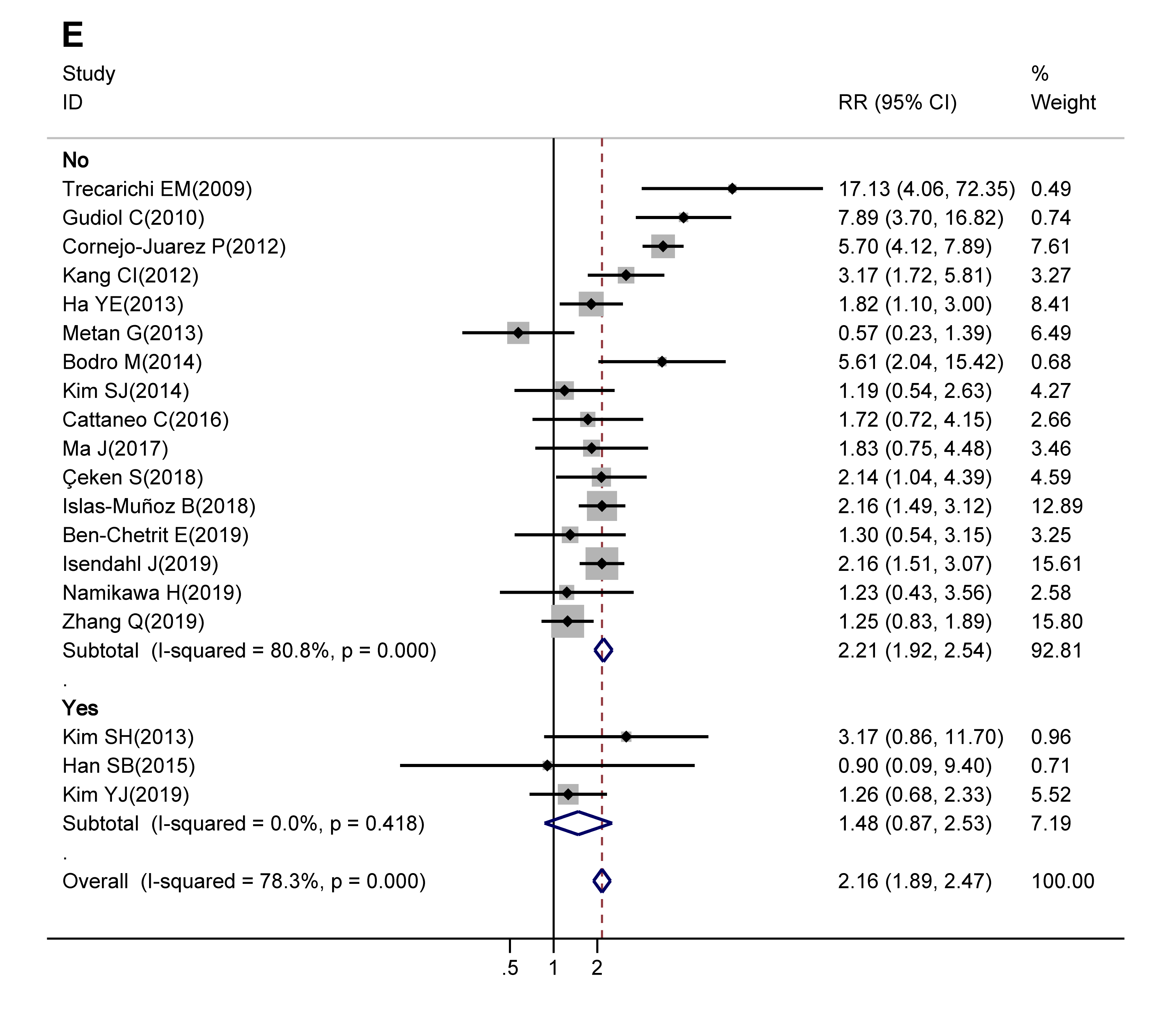


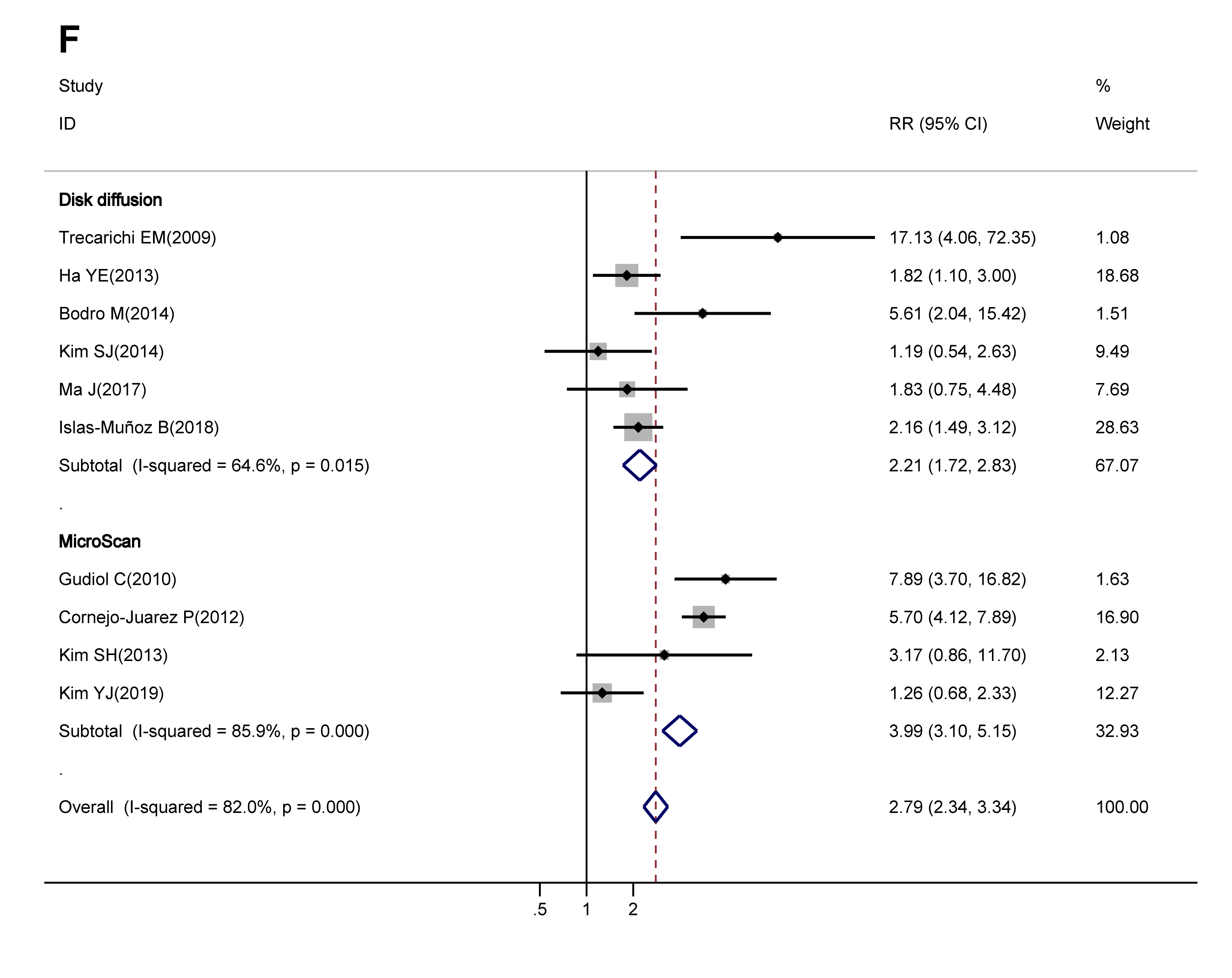


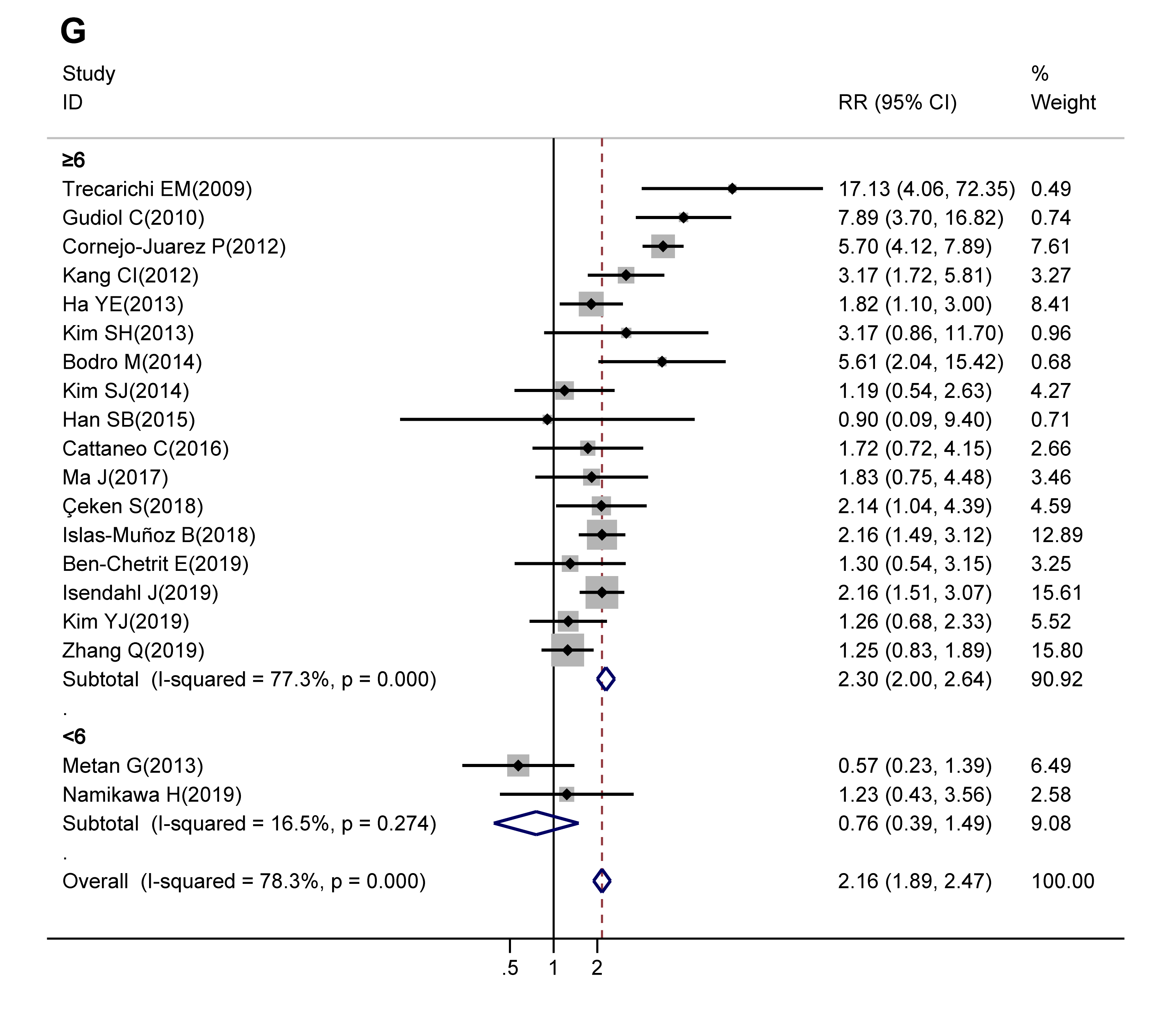


**Figure S1.** Forest plots of mortality in BSIs due to ESBL-PE among patients with malignancy by study design (**A**), region (**B**), population (**C**), malignancy type (**D**), FN (**E**), ESBL detection methods (**F**), NOS score (**G**). RR, relative risk; CI, confidence interval; BSIs, bloodstream infections; Weights are from random-effects analysis. The size of the squares is analogous to the study's weight. Diamonds represent the pooled RRs and their confidence interval.


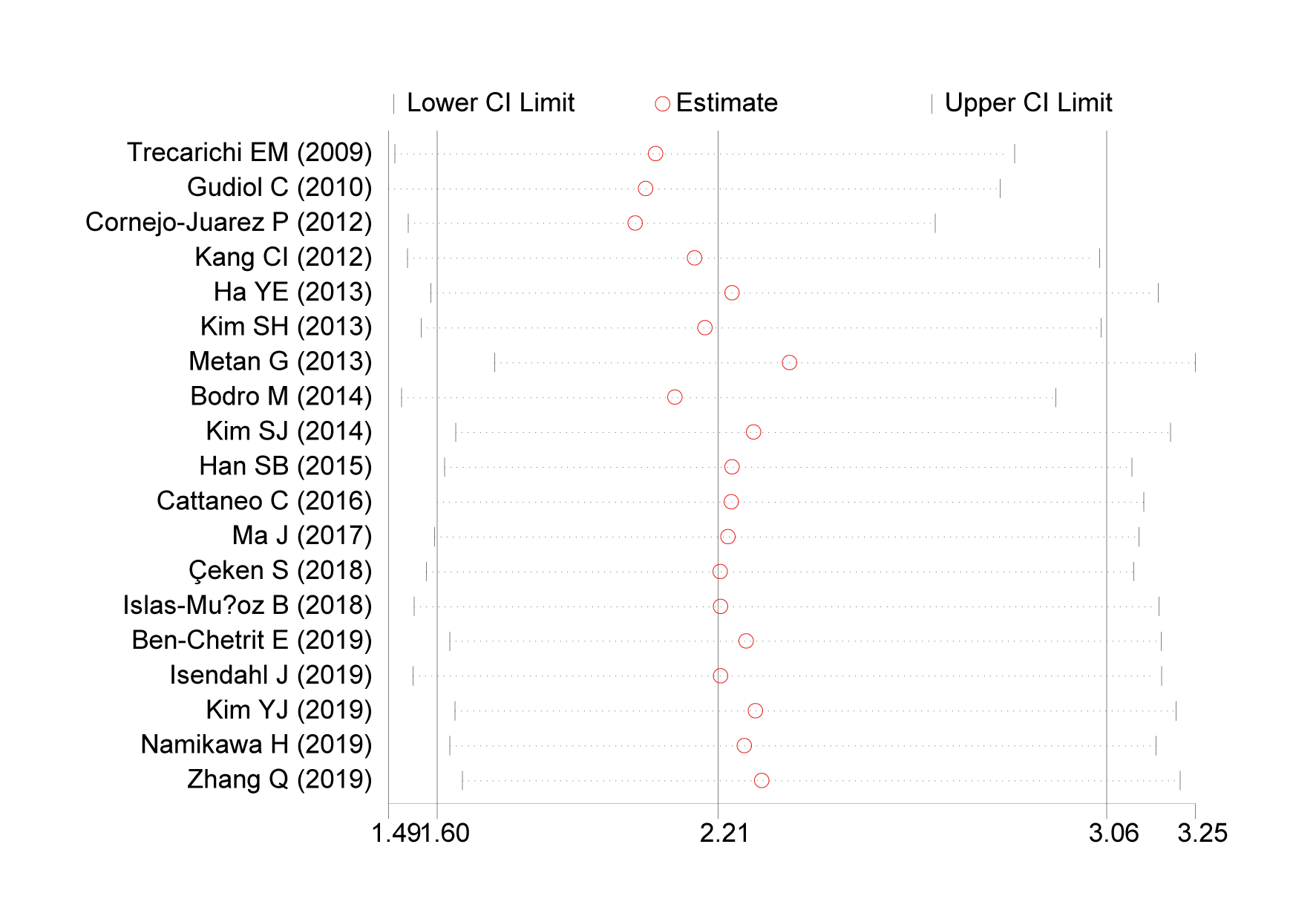


**Figure S2.** Sensitivity analysis of mortality in BSIs due to ESBL-PE among patients with malignancy.


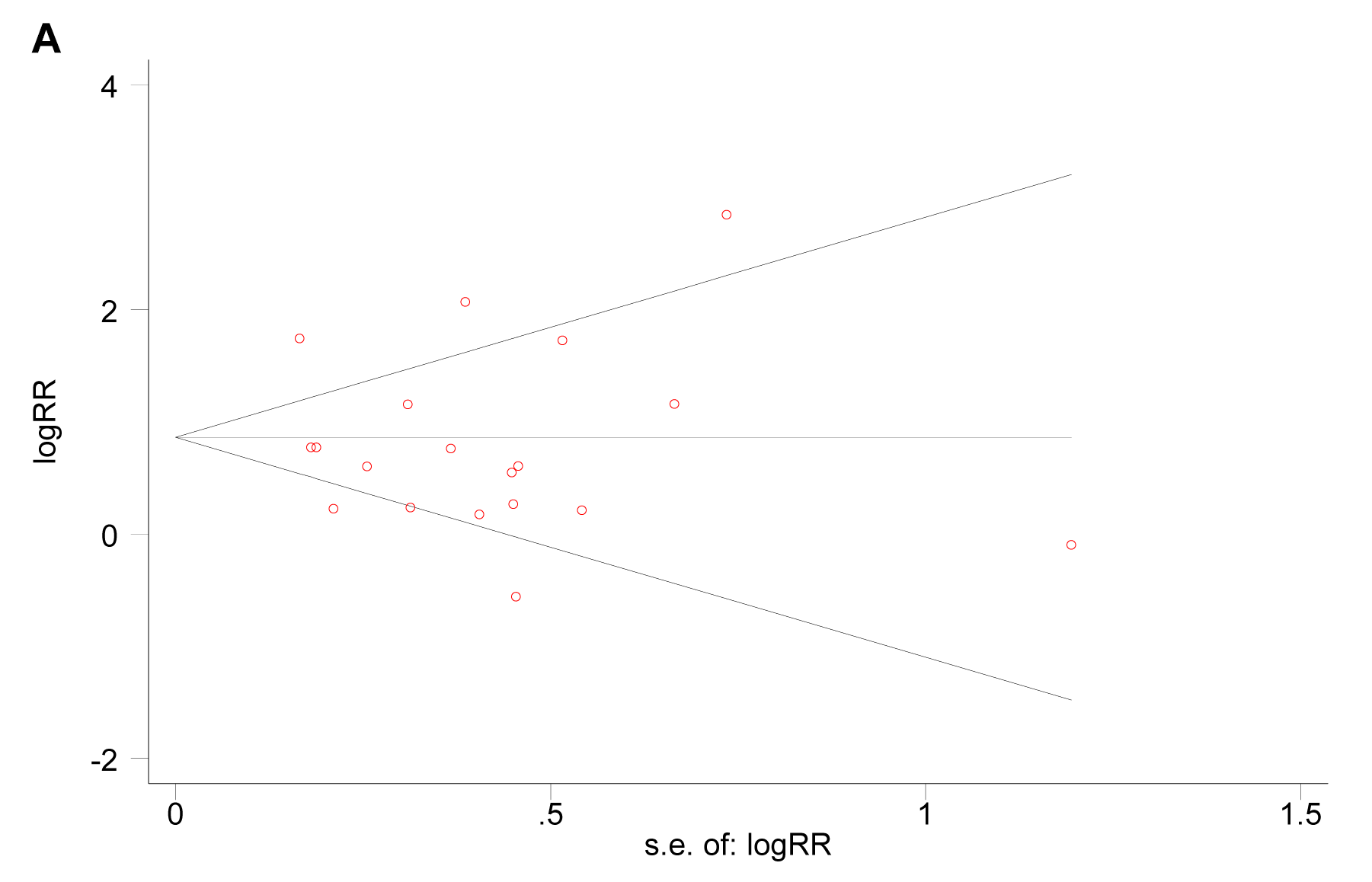


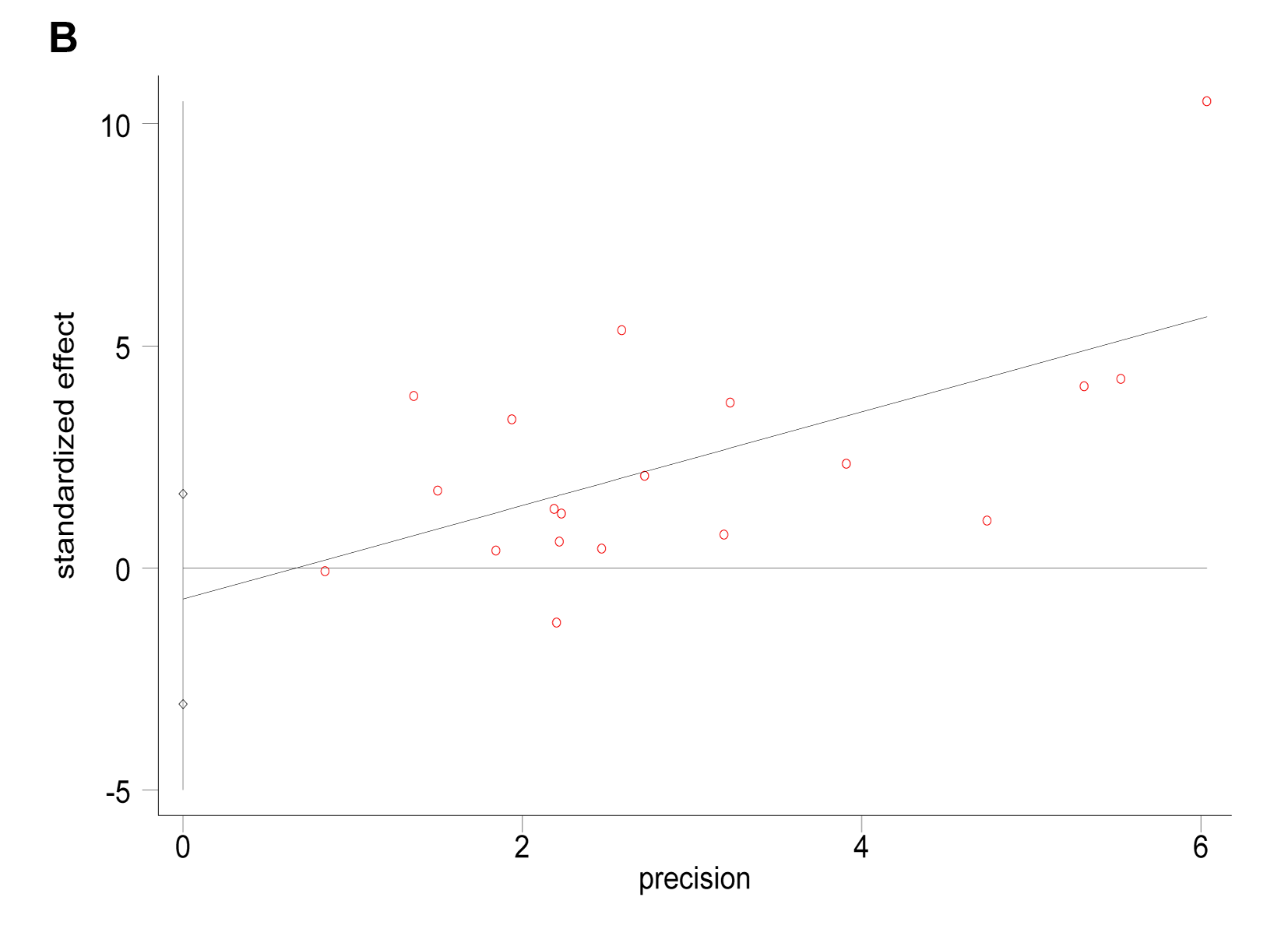


**Figure S3.** Tests for publication bias. (**A**) Begg's funnel plot with pseudo 95% confidence limits; (**B**) Egger's publication bias plot.
